# Mechanochemical Encapsulation of Caffeine in UiO-66 and UiO-66-NH_2_ to Obtain Polymeric Composites by Extrusion with Recycled Polyamide 6 or Polylactic Acid Biopolymer

**DOI:** 10.3390/polym16050637

**Published:** 2024-02-27

**Authors:** Cristina Pina-Vidal, Víctor Berned-Samatán, Elena Piera, Miguel Ángel Caballero, Carlos Téllez

**Affiliations:** 1 Instituto de Nanociencia y Materiales de Aragón (INMA), CSIC-Universidad de Zaragoza, 50009 Zaragoza, Spain; crispina@unizar.es (C.P.-V.); victorberned@unizar.es (V.B.-S.); 2 Chemical and Environmental Engineering Department, Universidad de Zaragoza, 50018 Zaragoza, Spain; 3 Research and Development Department, Nurel S.A., Ctra. Barcelona km 329, 50016 Zaragoza, Spain; epiera@samca.com (E.P.); acaballero@samca.com (M.Á.C.)

**Keywords:** metal organic framework, UiO-66, UiO-66-NH_2_, caffeine, microencapsulation, textile composite, polyamide, polylactic

## Abstract

The development of capsules with additives that can be added to polymers during extrusion processing can lead to advances in the manufacturing of textile fabrics with improved and durable properties. In this work, caffeine (CAF), which has anti-cellulite properties, has been encapsulated by liquid-assisted milling in zirconium-based metal–organic frameworks (MOFs) with different textural properties and chemical functionalization: commercial UiO-66, UiO-66 synthesized without solvents, and UiO-66-NH_2_ synthesized in ethanol. The CAF@MOF capsules obtained through the grinding procedure have been added during the extrusion process to recycled polyamide 6 (PA6) and to a biopolymer based on polylactic acid (PLA) to obtain a load of approximately 2.5 wt% of caffeine. The materials have been characterized by various techniques (XRD, NMR, TGA, FTIR, nitrogen sorption, UV–vis, SEM, and TEM) that confirm the caffeine encapsulation, the preservation of caffeine during the extrusion process, and the good contact between the polymer and the MOF. Studies of the capsules and PA6 polymer+capsules composites have shown that release is slower when caffeine is encapsulated than when it is free, and the textural properties of UiO-66 influence the release more prominently than the NH_2_ group. However, an interaction is established between the biopolymer PLA and caffeine that delays the release of the additive.

## 1. Introduction

In recent years, there has been a surge in industry interest in using microencapsulation processes to develop innovative materials. Microencapsulation has various benefits and advantages since active substances, such as vitamins, scents, essential oils, biocides, and drugs, may be encapsulated to preserve them from environmental factors, increase their temperature resistance, control the release rate of the additive encapsulated, or make liquids compatible with solids [1,2,3,4]. In the textile sector, microencapsulation offers many opportunities to improve the properties of textiles and provide new functionalities. Examples of functional textiles incorporating capsules include color-changing textiles [5], insect-repellent textiles [6], textiles for thermal control [7], antimicrobial [8,9] and medical textiles [10], and cosmetotextiles [11,12,13], among others. A wide range of methods and procedures have been developed to create capsules with desired materials and target properties: chemical methods (interfacial polymerization, in situ polymerization), physicochemical methods (complex coacervation, molecular inclusion), and physical procedures (spray drying, solvent evaporation) [14]. The capsules obtained through these procedures usually consist of a polymeric layer that covers the additive. An alternative to these capsules is to encapsulate additives in porous materials, both inorganic (e.g., zeolites [15]) and organic–inorganic materials (e.g., MOF [16]). The former allow processing at high temperatures while the latter preserve this property and are more compatible with textiles due to their organic–inorganic nature.

Metal–organic frameworks (MOFs) are porous crystalline hybrid materials made by linking a metal ion or cluster with organic linkers creating crystal lattices. These materials have several characteristics and properties that are of interest in many fields. They exhibit high porosity, high specific surface areas, excellent chemical and thermal stability, and the possibility of varying the pore size, shape, or chemical functionality by modifying the metal cluster and the nature of the linker. This makes them attractive for numerous applications, such as encapsulation [16], the development of selective membranes for molecular separation [17,18], adsorption and storage of gases [19], catalysis [20,21], and in biomedicine [22]. Among various MOF designs, those featuring zirconium-based structures incorporating [Zr_6_O_4_(OH)_4_]^12+^ cluster nodes and carboxylate linkers are highly appealing due to their outstanding chemical and thermal stability. In particular, UiO-66 stands out in this category. Comprising hexanuclear oxozirconium clusters and organic terephthalate ligands, it adopts a face-centered cubic (fcu) topology with a hydrophilic surface. Since its discovery in 2008, UiO-66 has become one of the most widely utilized MOFs [23]. Its popularity is attributed to its distinctive properties, such as ease of functionalization and tunability arising from its isoreticular structure [24]. This structural flexibility enables the creation of similar materials with the same UiO-66 topology by modifying either the metal cluster or the ligand. Consequently, a diverse range of materials with varying functionalities and pore sizes can be obtained. Notable among these isoreticular structures is the UiO-66-R, where R represents the functional group. Depending on the ligand used, the synthesized MOF can be denoted as UiO-66-NH_2_, UiO-66-NO_2_, or UiO-66-Br, among others. The pore aperture size of UiO-66 is theoretically estimated to be around 6.0 Å [23]. The remarkable thermal stability of UiO-66 is explained by the robust Zr-O bonds and the ability of the inner Zr_6_ cluster to undergo reversible rearrangement following the dihydroxylation or rehydration of the μ_3_-OH groups [25].

However, the preparation process of these materials mostly involves organic solvents, such as N,N-dimethylformamide (DMF) or N,N-dimethylacetamide (DMAC), which are highly toxic and hazardous to the environment and human health. Therefore, it is necessary to develop greener synthesis methods [26] in order to eliminate solvents entirely from the synthetic route or replace harsh organic solvents with water or less harmful solvents to reduce environmental costs. Some researchers have reported a green scalable modulated hydrothermal synthesis of UiO-66 in quite mild conditions using acetic acid as a modulator [27,28]. Other researchers have developed mechanochemical and solvent-free synthesis methods for UiO-66 and UiO-66-NH_2_ [29]. Mechanochemistry minimizes the use of organic solvents. This technique includes a process known as liquid-assisted grinding (LAG), in which a small amount of liquid is added [30,31].

Caffeine, known as 1,3,7-trimethylxanthine, is a versatile drug with amphiphilic properties that has gained significant attention in the cosmetic industry due to its anti-cellulite properties. There are studies in the literature in which caffeine is encapsulated in the MOFs [16,32,33,34] by immersing the previously synthesized MOF in a caffeine solution, which entails a subsequent step of filtration and drying. To improve this process, various advances have been made to limit the steps, such as performing the in situ synthesis of the MOF with caffeine [35,36] or performing pressure encapsulation with the already synthesized MOF [37]. Additionally, a solid-state mechanochemical strategy for additive encapsulation in MOF nanoparticles via a ball milling method has been reported [38]. The encapsulation occurs using a liquid-assisted grinding (LAG) method. This is an environmentally friendly process compared to the classic liquid phase encapsulation processes, since the use of solvent is minimized and the additive is fully used, reducing waste.

The incorporation of caffeine into polymer composites has been studied in diverse fields. Labay et al. analyzed the effect on caffeine release in plasma-treated polyamide (PA) samples [39,40] that had been impregnated with a caffeine solution. Sta et al. obtained caffeine-loaded poly(N-vinylcaprolactam-co-acrylic acid) composites by electrospinning which were evaluated as drug delivery systems [41]. Li et al. manufactured polyvinyl-alcohol (PVA) nanocomposites incorporating caffeine and riboflavin in the preparation by electrospinning for evaluating a fast-dissolving delivery system [42]. Similar to this study, caffeine and paracetamol were mixed with a polyvinylpyrrolidone (PVP) solution and nanocomposites were prepared by electrospinning [43]. Li et al. investigated the effects of caffeine on the degradation of the PLA matrix in caffeine delivery systems [44]. For cosmetic purposes, Tipduangta et al. developed cellulose acetate/PVP composites for caffeine delivery [45]. Recently, caffeine has been encapsulated in porous materials (Zeolite Y, ZIF-8, and MIL-53) and introduced during the spinning of polyamide 6 fibers for textile applications [16].

**Figure 1:**
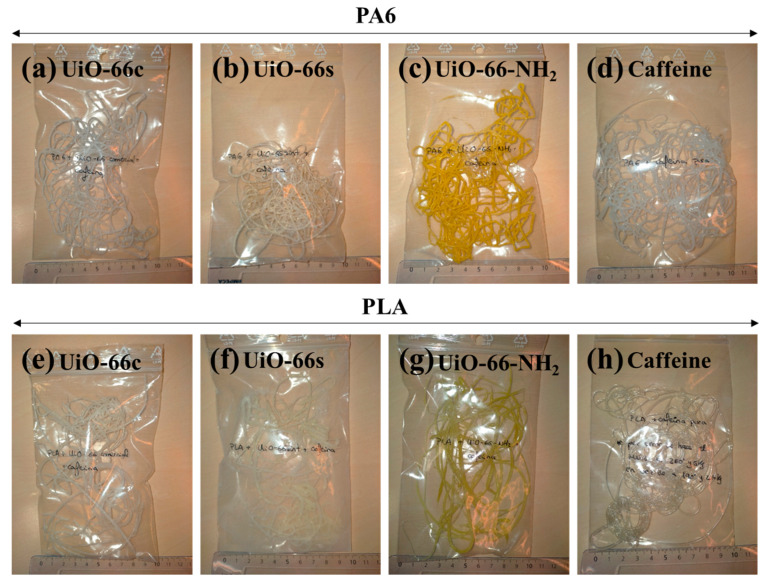
Photographs of the prepared polymer composites: (**a**) PA6 + CAF@UiO-66c, (**b**) PA6 + CAF@UiO-66s, (**c**) PA6 + UiO-66-NH_2_, (**d**) PA6 + caffeine, (**e**) PLA + CAF@UiO-66c, (**f**) PLA + CAF@UiO-66s, (**g**) PLA + CAF@UiO-66-NH_2_, (**h**) PLA + caffeine.

In the present work, caffeine-loaded UiO-66 capsules were developed using a ball mill-encapsulation method which minimizes the use of water and reduces waste. For this encapsulation process, two materials with different textural properties were compared: commercial UiO-66 and one synthesized by a solvent-free method. In addition, UiO-66 capsules were compared with the functionalized form, UiO-66-NH_2_, which was synthesized by a solvothermal synthesis in ethanol, avoiding the use of harmful solvents, such as DMF. Furthermore, the present study examined the use of more environmentally friendly processes and polymers by using recycled polyamide 6 (PA6) and polylactic acid (PLA), which is a biodegradable polymer used as an alternative to petroleum [46]. Then, the developed capsules were incorporated into PA6 and PLA composites during the extrusion process (see Figure 1). Therefore, the capsules remained embedded inside the composites improving caffeine durability and resistance, and consequently, the useful life of the final product. Traditionally, the addition of capsules to polymers for textile use has been performed in the last step and only superficially [47], so their durability is quite limited. The high temperatures used in the extrusion process must be taken into account, and UiO-66 capsules are suitable for resisting these temperatures.

## 2. Materials and Methods

### 2.1. Materials

Commercial UiO-66 (Strem Chemicals Inc., Newburyport, MA, USA) was purchased from CymitQuimica (Barcelona, Spain) and activated in an oven at 300 °C for 2 h. Zirconium (IV) oxychloride octahydrate (ZrOCl_2_·8H_2_O, Glentham Life Sciences, Corsham, UK, 98%) and tetraethylammonium bromide (TEABr, TCI chemicals, Tokyo, Japan, >98%) were also supplied by CymitQuimica. 1,4-benzenedicarboxylic acid (BDC, Aldrich, San Luis, MO, USA, 98%) and caffeine (1,3,7-trimethylxanthine, ReagentPlus, Aldrich) were purchased from Sigma-Aldrich (San Luis, MO, USA). Zirconium (IV) chloride (ZrCl_4_, Alfa Aesar, Haverhill, MA, USA, +99.5%), 2-aminoterephthalic acid (NH_2_-BDC, ThermoScientific, Waltham, MA, USA, 99%), and formic acid (HCOOH, FisherScientific, Waltham, MA, USA, 90%) were purchased from ThermoFisher Scientific (Waltham, MA, USA). As solvents, absolute ethanol (EtOH, 99.5%) and deionized water were provided by Productos Gilca S.C. (Zaragoza, Spain). Recycled Polyamide 6 (Recomyde^®^ B30 P4) and Biopolymer PLA based (Inzea^®^ F19) were provided by NUREL S.A. (Zaragoza, Spain). 

### 2.2. MOFs Synthesis

An efficient solvent-free method was used for the UiO-66 preparation following a procedure described in the literature, with slight modifications [48]. The synthetic procedure was as follows: A mixture of BDC (1 mmol), ZrOCl_2_·8H_2_O (1 mmol), and TEABr (1.42 mmol) was ground in a mortar for around 10 min until the mixture was homogeneous. It was then transferred to an autoclave and left at 190 °C for 24 h. After this time, the autoclave was left to cool down and the resultant solid was washed with warm EtOH (three or four times). Next, the solid was dried in an oven at 80 °C overnight. Finally, for MOF activation it was placed in a vacuum oven at 200 °C for 12 h. This method is easily scalable due to the absence of solvent, so, maintaining the proportions, several grams can be produced. This MOF is labeled UiO-66s to differentiate it from the commercial MOF labeled UiO-66c.

**Figure A1:**
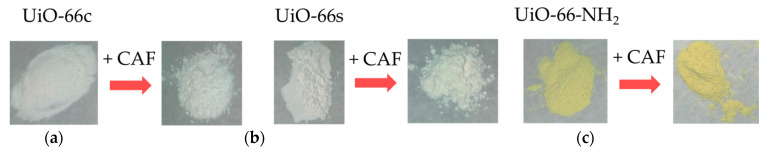
Images of (**a**) UiO-66c, (**b**) UiO-66s, and (**c**) UiO-66-NH_2_, before and after caffeine encapsulation.

The UiO-66-NH_2_ synthesis was carried out using a solvothermal method [49]. In this synthesis, the Zr metal was provided by ZrCl_4_ and the ligand in the amino-functionalized form (NH_2_-BDC). The procedure consisted of dissolving ZrCl_4_ (2 mmol) and NH_2_-BDC (1 mmol) in 20 mL of ethanol. Next, 25 mL of 30 wt% formic acid in water was added as an acid modulator. The mixed solution was placed in an ultrasound system for 10 min. Then, the solution was transferred to an autoclave and placed in an oven at 110 °C for 12 h. The solid was washed three to five times with absolute ethanol and water. Finally, the UiO-66-NH_2_ sample was activated in a vacuum oven at 200 °C for 12 h. It should be noted that the UiO-66-NH_2_ sample is yellow while the UiO-66 samples are white (Figure A1).

### 2.3. Mechanochemical Encapsulation Method

The encapsulation process was performed following a ball milling method, in particular through liquid-assisted grinding (LAG). In this technique, the reagents, previously physically mixed, were added into the grinding jar. Additionally, a small quantity of solvent, known as assisting liquid, was added to the solids. This may facilitate mechanochemical processes [31], help to obtain a better distribution of the solids, and enhance the diffusion, and consequently, the caffeine encapsulation. A Vibratory Micro Mill PULVERISETTE 0 (Fritsch, Idar-Oberstein, Germany) equipped with a mortar and a grinding ball, both made of stainless steel, was used. The operation conditions were optimized in terms of operation time, weight of solids, and amount of solvent. The procedure was the following: UiO-66 or UiO-66-NH_2_ (750 mg) and caffeine (250 mg) were physically mixed and added to the mortar. Then, 1 mL of distilled water was added as the assisting liquid, since caffeine presents a moderate water solubility (20 g/L at room temperature) and, thus, the presence of water will facilitate its diffusion within the pores of the UiO-66. After 2 h of ball mill operation, the loaded-caffeine MOF capsules, named CAF@MOF, were dried in an oven at 100 °C for 2 h to eliminate the water present in the samples. Figure A1 shows that the samples did not change color when the caffeine was encapsulated.

### 2.4. Preparation of Polymer Composites with UiO-66 Capsules with Caffeine

The CAF@MOF capsules were incorporated into the textile composites during the extrusion process. Two kinds of polymer were studied, polyamide (PA6) and polylactic acid (PLA). In each composite, the weight percentage of the caffeine-loaded capsules was 10 wt%, and, taking into account that the caffeine loading in the capsules was 25 wt%, the theoretical value of caffeine in the composites was 2.5 wt%. In order to determine the efficacy of the capsules, a composite was prepared with pure caffeine, without encapsulation in UiO-66. In this case, the amount of caffeine added in the extrusion process was proportional to the caffeine encapsulated in the capsules. Therefore, all the composites had the same quantity of theoretical caffeine. These composites were prepared using an Instron^®^’s Melt Flow Tester equipment model CEAST MF20 (Barcelona, Spain). The capsules were previously mixed with the polymer and this mixture was added to the equipment and heated. The PA6 composites were prepared at 260 °C while the PLA composites, given their lower melting temperature, were prepared at 190 °C. A weight (5 kg for PA6 composites and 2.16 kg for PLA composites) was placed on a piston which was located above the heated mixture in order for the melted polymer to flow through a hot nozzle, cooling at the outside of the oven and creating the extruded composites. Figure 1 shows images of the polymer composites with the UiO-66 capsules and caffeine. The samples have a whitish color except for the samples prepared with CAF@UiO-66-NH_2_, which are yellowish, consistent with the color of the synthesized MOFs (see Figure A1).

### 2.5. Characterization

The formation of UiO-66 was studied by X-ray diffraction (XRD) analysis using an Empyrean PANanlytical diffractometer (Malvern Panalytical, Malvern, UK) with a copper anode and a graphite monochromator to select CuKα radiation (λ = 1.5406 Å). Data were collected in the 2.5–40° 2θ range with a scanning rate of 0.01 °/s.

The textural properties, including BET surface areas with pore volumes, were measured using a Micromeritics TriStar 3000 (Micromeritics Instrument Corporation, Norcross, GA, USA) with previous degasification at 200 °C (for UiO-66) and at 175 °C (for UiO-66-NH_2_) for 10 h with a heating rate of 10 °C/min in a Micromeritics VacPrep™ 061 (Micromeritics Instrument Corporation, Norcross, GA, USA).

Thermogravimetric analysis (TGA) was performed using Mettler Toledo TGA/SDTA 854e equipment (Mettler Toledo, Columbus, OH, USA). Samples placed in 70 µL alumina pans were heated up to 700 °C with a heating rate of 10 °C/min. 

Attenuated total reflection-Fourier transformed infrared spectroscopy (ATR-FTIR) was carried out in a Bruker Vertex 70 FTIR spectrometer (Bruker, Billerica, MA, USA) with a DTGS detector and a Golden Gate diamond ATR accessory. The spectra were recorded by averaging 40 scans in the 600–4000 cm^−1^ wavenumber range with a resolution of 4 cm^−1^.

The surface morphology of the samples and their particle sizes were studied by SEM. Moreover, SEM-EDX analysis was performed on the polymer composites in order to determine the atomic percentage at different points and to confirm the presence of UiO-66. The samples were cut under a liquid nitrogen atmosphere and their cross sections were viewed through electron microscopy using an FEI-Inspect F50 microscope at a voltage of 10 kV. The samples were previously coated with palladium under vacuum conditions.

The powder samples were also observed by Transmission Electron Microscopy (TEM) using a FEI Tecnai T20 microscope (FEI Company, Hillsboro, OR, USA) operated at 200 kV accelerating voltage. The samples were dispersed in ethanol and drops of the solution were placed onto a carbon-coated grid.

Caffeine release from the CAF@UiO-66 samples was evaluated at 25 °C using distilled water. Suspensions of 10 mg CAF@MOF samples in 100 mL of water were prepared and the concentration of caffeine in each 3 mL aliquot was determined using a calibration curve. Each release experiment was performed twice. In order to achieve a gradual release, these experiments were carried out without stirring. Additionally, this solid–liquid extraction was performed with a physical mixture of MOF and caffeine in the same ratio as the ball mill samples (25 wt% of caffeine). Caffeine release from the polymer composites was carried out similarly. Around 500 mg of composites were cut into similar pieces and added to 100 mL of distilled water. Periodically, a liquid sample (3 mL) was taken from the release media. The initial temperature was 25 °C, which was increased to 50 °C, and then to 80 °C in order to achieve a gradual release over time. The amount of caffeine in the liquids obtained from solid–liquid extraction was quantified by ultraviolet spectroscopy (UV–Vis) using a JASCO V-670 spectrophotometer (JASCO International, Tokyo, Japan) using water as a solvent at a wavelength of 272 nm.

Solid-state cross-polarization magic angle spinning (CP MAS) ^13^C-Nuclear Magnetic Resonance (NMR) spectra were acquired with a Bruker Avance III 400 MHz Wide Bore spectrometer (Bruker, Billerica, MA, USA) equipped with a 4 mm CP MAS 1H-BB probe. Approximately 100 mg of the product was packed in a 4 mm zirconium rotor and sealed with a Kel-F cap. The ^13^C CP spectra were acquired with a MAS rate of 10 kHz, a ramp-CP contact time of 3 ms, and a 7 s recycle delay. For the ^1^H NMR measurement, the liquid samples taken from the release experiments were evaporated and dispersed in deuterated water. NMR spectra were acquired with a Bruker Neo 500 MHz spectrometer equipped with a 5 mm iProbe 1H-BB at 25 °C using TMS as the internal standard. The ^1^H and ^1^H presaturation experiments were conducted with 64 scans.

During the preparation of the polymer composites (previously explained in Section 2.4), the equipment used also recorded their fluidity values in accordance with the ISO 1133 standard (reference [50]) test method. The equipment measured the fluidity at regular intervals, obtaining an average value at the end of the process. The results of the measurements, known as the melt volume rate (MVRs), are expressed in units of cm^3^/10 min.

## 3. Results and Discussion

### 3.1. MOF Characterization

**Figure 2:**
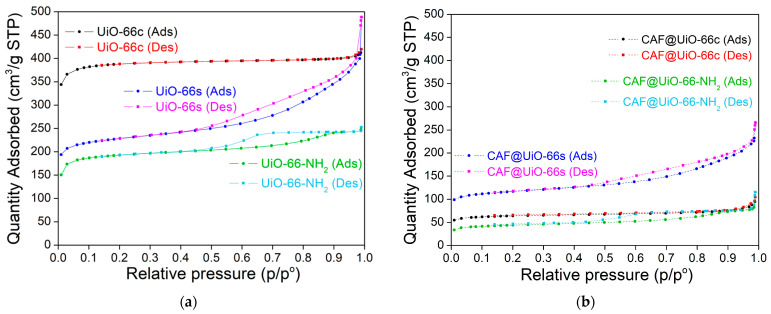
Nitrogen adsorption–desorption (Ads-Des) isotherms of UiO-66c, UiO-66s, and UiO-66-NH_2_ before (**a**) and after (**b**) caffeine encapsulation.

To evaluate the encapsulation efficiency in porous materials, various MOF samples were used in this work. Two types of UiO-66 with different textural properties, and the amino-functionalized form UiO-66-NH_2_, were tested. Therefore, first of all, the MOF samples were characterized by nitrogen adsorption–desorption (Figure 2 and Table 1).

The different textural properties of UiO-66c and UiO-66s can be seen from the sorption isotherms represented in Figure 2. For UiO-66c, the isotherm is a type-I according to the IUPAC classification, indicating a microporous structure of this material (see also micropore volume in Table 1). For the synthesized MOFs (UiO-66 and UiO-66-NH_2_), the isotherms are a combination of type I and type IV, indicating the presence of micropores and mesopores, which can influence caffeine encapsulation and its posterior gradual release. Furthermore, the synthesized MOFs that have a smaller size, as seen below, show an increase in nitrogen adsorption at values close to P/P_0_ ≈ 1, which is related to the condensation between nanoparticles. In the literature, the reported BET surface areas for UiO-66 range from ~600 to 1800 m^2^/g [51]. The surface area values found here for UiO-66 are in that order. The lower BET surface values of UiO-66s (771 m^2^/g) compared to UiO-66c (1298 m^2^/g) are in agreement, as will be seen below, with the XRD pattern of this material, which was not as crystalline as UiO-66c. The BET surface area (650 m^2^/g) and the micro-pore volume (0.240 cm^3^/g) of UiO-66-NH_2_ were slightly lower than those obtained for UiO-66s (771 m^2^/g and 0.271 cm^3^/g). This slight decrease could be related to the presence of the amino group that reduces the space in the pores. In any case, the surface area for UiO-66-NH_2_ is within the range of values reported in the literature, ~630–996 m^2^/g [25,30].

**Figure 3:**
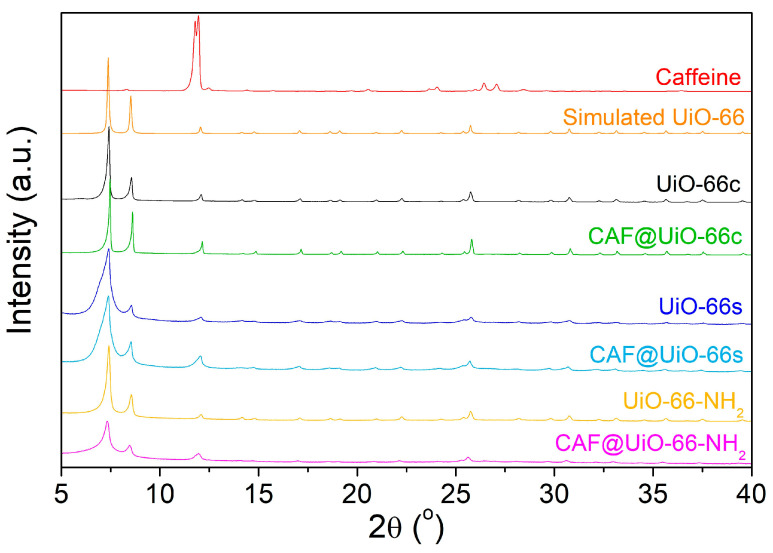
XRD spectra of UiO-66s, UiO-66c, UiO-66-NH_2_, and simulated UiO-66 using Mercury 3.8 software [54] and CCDC 1018045 [55] from Cambridge Crystallographic Data Centre. The XRD of the samples after the caffeine encapsulation process are also shown.

Figure 3 shows the XRD spectra obtained after the activation of UiO-66 synthesized using a green solventless method, UiO-66-NH_2_ synthesized using a solvothermal method, UiO-66c, and simulated UiO-66. The XRD pattern of the synthesized UiO-66 is in line with the simulated pattern. The main peaks at approximately 2·θ = 7.4, 8.5, and 25.7° are related to the crystallographic planes (111), (002), and (224) [52], and affirm the successful synthesis of the desired MOF structure. The agreement of peaks in the XRD shows that UiO-66-NH_2_ was also properly synthesized by the solvothermal method. It was noted that UiO-66 and UiO-66-NH_2_ displayed comparable diffraction patterns, suggesting that ligand functionalization had no impact on the MOF crystal structure [53]. In the case of the UiO-66s, the peaks are broader than those of the UiO-66c, which may indicate a lower degree of crystallinity and also a smaller crystal size, as explained in the microscopy study below.

The average crystal size (D) was calculated by Scherrer’s equation (Equation (1)) using the peaks at 7.4, 8.5, and 25.7° for the three MOF samples.

(1) $$D=\frac{K\;\cdot \;\lambda }{\beta \;\cdot \;\cos\theta }$$

The values obtained with the highest intensity peak at 7.4° were 49.5 nm, 13.4 nm, and 40.2 nm for the UiO-66c, UiO-66s, and UiO-66-NH_2_, respectively. The calculated values for the other peaks are similar to these previous values (see Table A1). In the subsequent sections, the trend of these values will be corroborated by observing SEM images. The highest particle size was obtained for UiO-66c and the lowest for UiO-66s.

**Figure 4:**
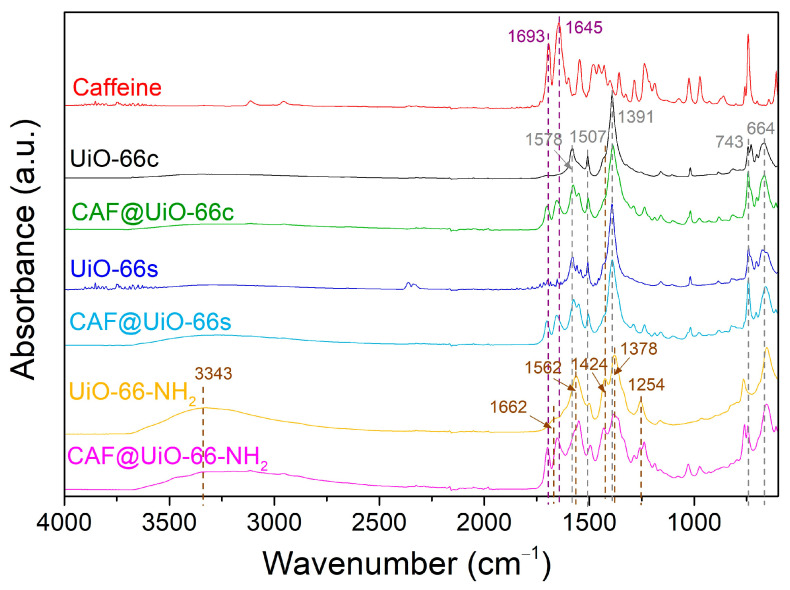
FTIR spectra of UiO-66c, UiO-66s, and UiO-66-NH_2_ before and after caffeine encapsulation.

The FTIR spectra (Figure 4) of the UiO-66c and the synthesized MOFs are compared to confirm the MOF synthesis and identify the main molecular groups. As can be seen, the synthesized UiO-66 presents a similar pattern to that of the UiO-66c. In particular, the band at 1578 cm^−1^ is assigned to the C=O carbonyl bond stretching vibration of the carboxyl group. The weak band at 1507 cm^−1^ is related to the C=C vibration of the benzene ring. The strong band at 1391 cm^−1^ is ascribed to the C-O bond of the C-OH group of the carboxylic acid [56]. Next to this peak appears a small peak at 1424 cm^−1^ related to the C-C of the benzene ring. Other typical bands for UiO-66 are at 743 and 664 cm^−1^ for the symmetric and asymmetric stretching of the O-Zr-O bonds, respectively [57]. In the case of UiO-66-NH_2,_ the bands that appear relate to the functional groups corresponding to the amino group. There is a broad absorption band at around 3343 cm^−1^ that corresponds with the symmetric and asymmetric vibration bands of –NH_2_. The small peak at 1662 cm^−1^ corresponds to the N-H bending vibration [58]. The band at 1562 cm^−1^ is attributed to the C=O bond in the carboxyl group. The band at 1424 cm^−1^, characteristic of C-C in the benzene ring, is more pronounced than in the UiO-66 sample. Additionally, two bands are observed at 1254 and 1378 cm^−1^ corresponding to the band of C-N bonded between aromatic carbon and nitrogen [49].

**Figure 5:**
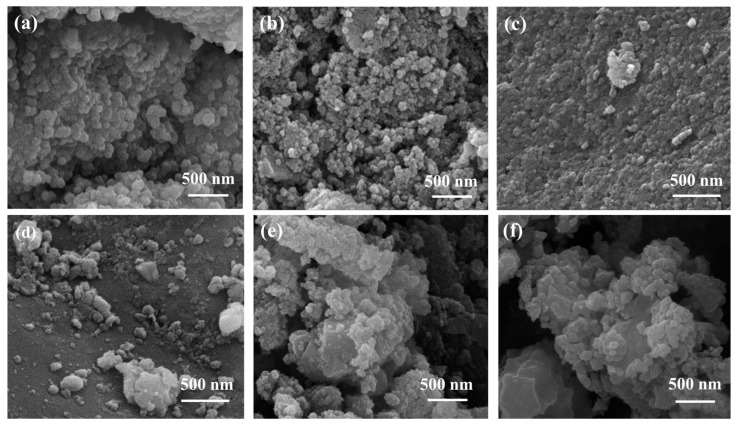
SEM images of (**a**) UiO-66c, (**b**) UiO-66s, and (**c**) UiO-66-NH_2_. SEM images of UiO-66 with encapsulated caffeine (**d**) CAF@UiO-66c, (**e**) CAF@UiO-66s, and (**f**) CAF@UiO-66-NH_2_.

**Figure A2:**
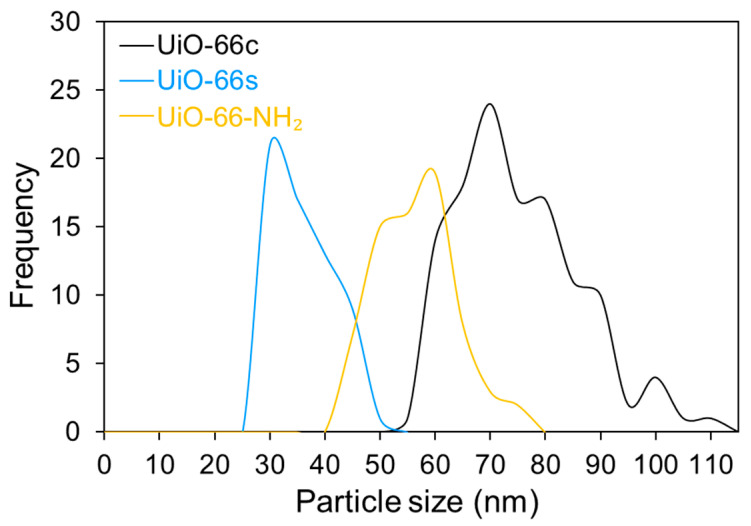
Particle size distribution of UiO-66c, UiO-66s, and UiO-66-NH_2_.

SEM images of the nanoparticles are shown in Figure 5. In this figure, it can be seen that the nanoparticles are relatively homogeneous, and the geometry is not fully developed, presenting an appearance between cubic and spherical. The particle size of these samples was measured using the ImageJ software [59], obtaining mean values of 77 ± 12 nm, 38 ± 5 nm, and 59 ± 7 nm for UiO-66c, UiO-66s and UiO-66-NH_2_, respectively. The size distribution of the MOF samples was calculated from these SEM images (Figure A2). As can be observed, the MOF samples present a uniform particle size distribution, having a maximum of around 70 nm, 30 nm, and 60 nm for UiO-66c, UiO-66s, and UiO-66-NH_2_, respectively, which are very close to the mean values.

The smaller particle size observed for the synthesized UiO-66 is in agreement with the lower crystallinity observed by XRD. In addition, the particle sizes observed by SEM analysis are similar to the values obtained by Scherrer’s equation (see Table A1). Using the highest intensity XRD peak, for UiO-66c the difference between values from the SEM and Scherrer’s equation is from 77 to 54 nm, for UiO-66s it is from 13.4 to 38 nm, and for UiO-66-NH_2_ from 59 to 43 nm. The difference is more pronounced in the case of the UiO-66s. This may be because the peak at 7.4° for the Scherrer’s equation calculation is more amorphous in the synthesized MOF sample than in the other samples (UiO-66c and UiO-66-NH_2_). In any case, the trends in particle size are predicted both by SEM and by the calculations made with Scherrer’s equation for any of the peaks used.

**Figure A3:**
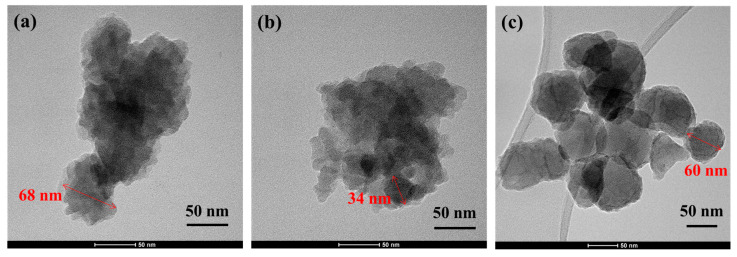
TEM images of (**a**) UiO-66c, (**b**) UiO-66s, and (**c**) UiO-66-NH_2_.

In order to examine these MOF samples in more detail, they were also characterized by TEM. Figure A3 shows that the nanoparticles in the UiO-66-NH_2_ sample were better dispersed than in the UiO-66 samples, which appeared more agglomerated, so the particle sizes in the latter were not as clear. The particle sizes obtained from this technique were similar to the values determined by SEM analysis.

**Figure 6:**
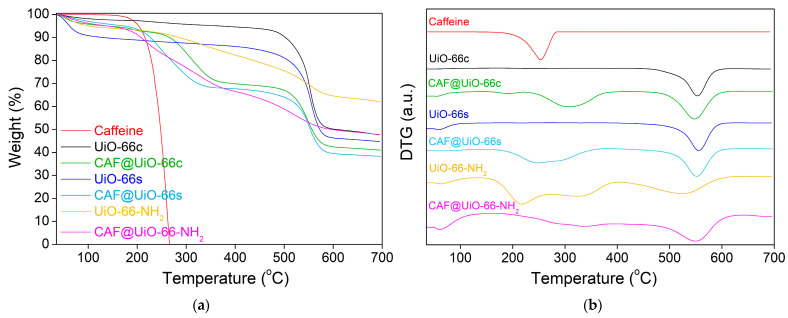
TGA (**a**) and DTG (**b**) analysis of UiO-66c, UiO-66s, and UiO-66-NH_2_ samples before and after caffeine encapsulation. TGA was carried out under nitrogen flow.

The thermal stability of the samples was tested by thermogravimetric analysis (TGA). Figure 6 shows the weight loss of the samples under the flow of nitrogen. The initial weight loss (until 100 °C) of the MOF samples corresponds to the removal of moisture from the samples. UiO-66c and UiO-66s presented high thermal stability (up to 500 °C), as reported in the literature [34]. This weight loss is related to ligand decomposition under pyrolytic conditions and the subsequent amorphization of the structure and pore collapse. The synthesized sample showed a higher content of moisture. The thermal stability of the UiO-66-NH_2_ was lower than that of the pristine UiO-66, because of the presence of an additional functional group [25], showing a weight loss from 300 to nearly 600 °C, which was more gradual than that of the UiO-66. After TGA under N_2_ flow, the residue originated from the carbonization of the ligand and the formation of ZrO_2_ coming from Zr and O elements in the UiO-66 [60].

### 3.2. Caffeine-Loaded Capsules

The XRD patterns of the UiO-66 samples before and after caffeine encapsulation are shown in Figure 3. Peaks corresponding to caffeine do not appear in the CAF@MOF samples, which indicates that there were no caffeine crystals in the samples, suggesting the encapsulation of the additive in the porous framework of UiO-66 and UiO-66-NH_2_. Slight shifts in the peaks can be noted after caffeine encapsulation. Particularly in CAF@UiO-66-NH_2_, the XRD peaks were displaced to a lower angle. This shift observed may involve an increase in the d-spacing or the distance between crystal planes in the UiO-66 structure. In the bibliography, the introduction of ionic liquids into the micropores of UiO-66 resulted in a slight expansion of the crystal lattice [61]. Nevertheless, the preservation of the XRD peaks indicates that the crystal structure remains after caffeine loading.

The presence of caffeine in the encapsulated samples was affirmed by using FTIR spectroscopy, as shown in Figure 4. In the spectra of caffeine, some typical bands were identified at 1693 and 1645 cm^−1^, corresponding to the carbonyl bond C=O. Slight shifts in carbonyl groups were detected in the spectra of CAF@UiO-66c (from 1693 and 1645 cm^−1^ to 1701 and 1653 cm^−1^), in the spectra of CAF@UiO-66s (to 1701 and 1654 cm^−1^), and in the spectra of CAF@UiO-66-NH_2_ (to 1698 and 1652 cm^−1^). These shifts suggest interactions between caffeine molecules and UiO-66 groups [34]. In the FTIR spectra, the main functional groups in the MOF are shown to have been preserved in the CAF@UiO-66 samples.

The thermal stability of the capsules was measured by using TGA. Additionally, with this technique, the caffeine encapsulation was estimated and was later corroborated by the release experiments and analyzed by UV–Vis. Therefore, the efficiency of additive encapsulation can be observed in terms of the initial degradation temperature of caffeine in the encapsulated samples. Figure 6 represents the TGA and DTG (derivative thermogravimetry) for the caffeine and UiO-66 samples before and after encapsulation.

For the CAF@UiO-66c sample, the biggest weight loss related to caffeine appears delayed (maximum weight loss at 310 °C and degradation between 220 and 380 °C) compared to the curve of pure caffeine (maximum weight loss at 250 °C and degradation between 180 and 290 °C). This delay in the caffeine degradation may be explained by its encapsulation in the UiO-66 framework. The percentage of encapsulated caffeine measured for this peak is 24.2 wt%. There is also a small weight loss (approximately 1.5 wt%) between 180 and 220 °C that may be related to free caffeine or caffeine superficially adsorbed on the MOF. The total value of caffeine present in this sample is close to the theoretical value added in the encapsulation process, which is 25 wt%. The MOF decomposition occurs at the same temperature as it occurs in the crystalline MOF before encapsulation; therefore, the UiO-66 structure remains unaffected after the grinding encapsulation process.

In the case of CAF@UiO-66s, the delay related to caffeine degradation is lower than for CAF@UiO-66c. In this case, the degradation of caffeine takes place between 180 °C and 370 °C with two maximums, one at approximately 240 °C and the other at 290 °C. This fact can be explained by the lower surface area of UiO-66s, which is not as crystalline as UiO-66c, and, therefore, the caffeine encapsulated inside the micropores will be less, and a part of the caffeine is adsorbed in mesopores or superficially adsorbed in the MOF since its external area is high given its small particle size. The caffeine load of this sample was 26.4 wt%, again, close to the theoretical value of 25 wt%.

In the case of CAF@UiO-66-NH_2_, the degradation of caffeine takes place between 180 °C and 380 °C with two maximums, one at approximately 230 °C and the other at 320 °C, which again can be related to two types of caffeine encapsulated in the micropores and the mesopores/superficial. Analyzing the weight loss corresponding to caffeine for the UiO-66-NH_2_ sample the additive load was 25.8 wt%.

The sorption isotherms before and after encapsulation have similar shapes for each MOF, although the quantity adsorbed in the encapsulated samples is much lower (Figure 2). The BET analysis was performed after the caffeine encapsulation (Table 1) in order to verify the decrease in the surface area resulting from the additive encapsulation. The specific surface area of UiO-66c decreased by 83% (from 1298 m^2^/g to 216 m^2^/g) while the decrease in UiO-66s was slightly lower at 49% (from 771 m^2^/g to 396 m^2^/g). Following the same trend, UiO-66-NH_2_ decreased its area by 77% (from 650 m^2^/g to 150 m^2^/g).

**Figure 7:**
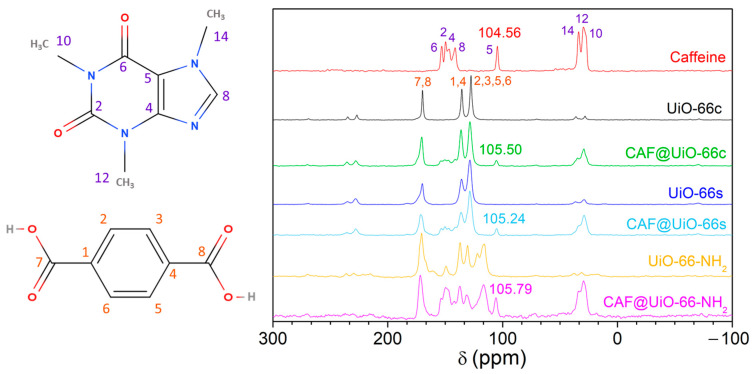
^13^C CP MAS NMR spectra of caffeine, UiO-66c, UiO-66s, and UiO-66-NH_2_, and the corresponding encapsulated samples (CAF@UiO-66c, CAF@UiO-66s, and CAF@UiO-66-NH_2_).

The ^13^C CP MAS NMR spectra (Figure 7) were achieved for pure caffeine, both UiO-66, UiO-66-NH_2_, and the caffeine encapsulated samples (CAF@UiO-66c, CAF@UiO-66s and CAF@UiO-66-NH_2_). The observed chemical shifts (in ppm) for caffeine correspond to 27.8 (C10), 29.61 (C12), 33.82 (C14), 104.56 (C5), 141.40 (C8), 146.55 (C4), 149.65 (C2), and 153.01 (C6) [62]. The NMR spectra of UiO-66 contain three peaks located at isotropic chemical shifts of 127.52 ppm (C2, C3, C5, C6), 135.53 ppm (C1, C4), and 169.78 ppm (C7, C8). The peak at the high chemical shift corresponds to carbon atoms from a carboxylic group. The peak at 127.52 ppm is assigned to carbon atoms bonded to a proton. The peak at 135.53 ppm corresponds to quaternary aromatic carbon atoms [63]. Peaks corresponding to pure caffeine are clearly seen in the encapsulated samples, in C5 105.50, 105.24, and 105.79 ppm for CAF@UiO-66c, CAF@UiO-66s, and CAF@UiO-66-NH_2_, respectively, with a slight change in chemical shift as compared with the caffeine spectra (104.56 ppm), suggesting the encapsulation of the additive. The intensity of these peaks is lower for encapsulated samples. In addition, the peaks of UiO-66 were observed in the ball mill samples, indicating the preservation of the UiO-66 structure.

**Figure A4:**
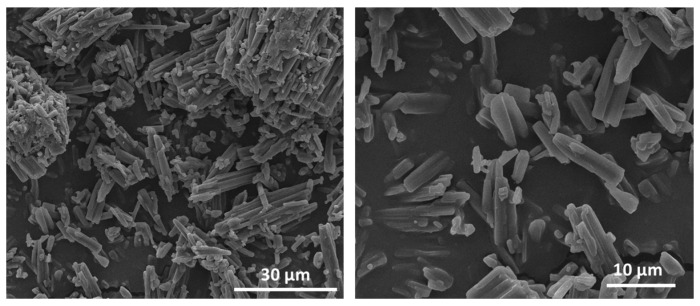
SEM images of caffeine crystals.

SEM analysis was used to observe the physical morphology of the samples after caffeine encapsulation (Figure 5). As can be observed, the UiO-66 morphology of all the samples was preserved after ball mill encapsulation; therefore, the crystalline framework and, by extension, most of the UiO-66 properties were maintained after the process. A pure caffeine sample was also seen using SEM (Figure A4). Caffeine crystals have an elongated shape with a particle size of approximately 7.8 ± 3.3 μm. This caffeine crystals do not appear in the encapsulated samples.

To summarize the characterization of the capsules with caffeine encapsulated in MOF by ball milling, the following indications ensure the correct encapsulation of caffeine: the XRD patterns do not show peaks corresponding to pure caffeine; the TGA analysis shows a delayed degradation of caffeine compared to pure caffeine and whose weight loss approximately coincides with the expected load of 25 wt% of additive; the FTIR and NMR analyses show the characteristic peaks of caffeine with slight shifts; the specific surface and pore volume of the capsules decrease significantly compared to the pure MOF; and the SEM images preserve the shape of the MOF without observing caffeine crystals. These indications are corroborated by the slower release of caffeine from the capsules than caffeine without encapsulating, as described in the caffeine-release section below.

### 3.3. Characterization of Textile Composites

**Figure 8:**
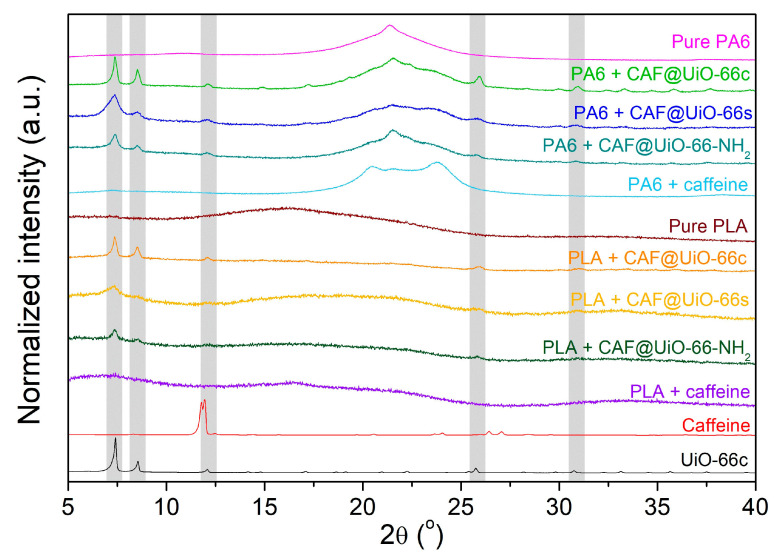
XRD of PA6 and PLA composites of UiO-66 encapsulated samples and pure caffeine.

Figure 8 represents the XRD spectra of the PA6 and PLA composites with capsules and with pure caffeine. The pure polyamide shows a wide peak around 2θ = 21.4°, characteristic of γ-PA6 [64], which indicates its semi-crystalline character. With the presence of capsules or caffeine, three peaks are related to PA6, the one just indicated above at 2θ = 21.4°, and two new peaks at 2θ = 20.4° and 23.7° related to α-PA6 [64,65]. This would indicate a certain degree of change in the crystalline character of PA6 with the presence of the filler. The pure PLA shows a practically amorphous character with a very wide peak centered at 2θ = 16° that seems to vary with the addition of capsules based on caffeine or pure caffeine, which would suggest a certain degree of interaction of these materials with the PLA polymer. According to the XRD results, some characteristic peaks of the UiO-66 are present in the sample composites with capsules, indicating the presence of the capsules and providing evidence that the crystalline structure of the MOF was maintained after the extrusion process. For polymer composites with synthesized UiO-66, the observed peaks are broader, as occurred in the XRD of the solid samples (see Figure 3). In the case of the PA6 and PLA composites with pure caffeine, there are no visible peaks corresponding to this additive.

**Figure 9:**
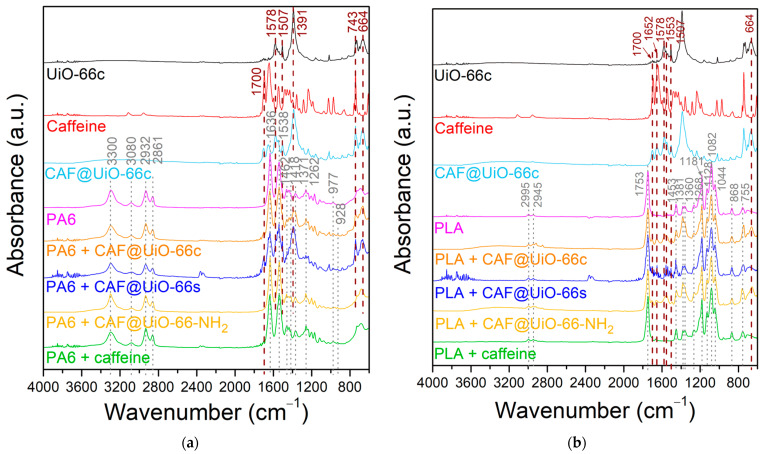
FTIR spectra of polymer composites of UiO-66 encapsulated samples and pure caffeine: (**a**) PA6. (**b**) PLA.

In addition, the presence of caffeine and UiO-66 in the polymer composites was studied by FTIR analysis. Figure 9 shows the spectra of the PA6 and PLA composites. In this figure, signals appear corresponding to the caffeine-loaded capsules and to each polymer. For the pure PA6 composite, the main bands of this polymer can be observed [66]. The bands located at 3300 and 1538 cm^−1^ were assigned to the stretching and bending vibration of the hydrogen bonds in the amide II. These same bonds for amide I present a band at 1636 cm^−1^. The carbonyl group (C=O) is represented by the band at 1636 cm^−1^. Additionally, the signal at 3080 cm^−1^ corresponds to the intramolecular bond between amide and carbonyl groups (-CONH_2_). The O=C-N bond related to the ring opening is confirmed by the presence of the band at 1262 cm^−1^ which corresponds to the C_aliphatic_-N bond. The bands observed at 2932 and 2861 cm^−1^ were assigned to the vibration of symmetrical and asymmetrical stretching of –CH_2_ and –CH_3_ groups, respectively. Additionally, the presence of groups was confirmed due to the presence of the bands at 1462 and 1418 cm^−1^ for –CH_2_, and the band at 1371 cm^−1^ corresponding to the –CH_3_ group. Finally, the γ-PA6 phase is denoted by the presence of a band at 977 cm^−1^ [64]. For the PA6 composites with capsules or caffeine, a band appears at 928 cm^1^ related to the α-PA6 phase. In Figure 9, the most important bands of UiO-66 at 1578 cm^−1^ (C=O carbonyl bond), 1507 cm^−1^ (C=C phenyl ring), 1391 cm^−1^ (C-O of carboxylic group), and 743 and 664 cm^−1^ (O-Zr-O) are reflected in the PA6 composites. These bands are marked by dark dash lines in Figure 9. Likewise, there are some bands that could correspond to caffeine, such as 1700 cm^−1^, and, although it coincides with a UiO-66 band, that of 743 cm^−1^.

The FTIR spectra of the pure PLA composites show the main bands for this polymer (Figure 9b) [67]. The bands located at 2995 and 2945 cm^−1^ correspond to the vibration of the symmetrical and asymmetrical stretching of C-CH_3_. The stretch of the carbonyl bond (C=O) is represented by the band at 1753 cm^−1^. The band located at 1453 cm^−1^ is due to the asymmetrical bending of the CH_3_ group. Additionally, the bands observed at 1381 and 1360 cm^−1^ represent the asymmetrical and symmetrical bending of CH. The bending of C=O is denoted by the presence of a band at 1268 cm^−1^. The stretching symmetrical vibration of the C-O-C bond corresponds to the bands at 1181, 1128, and 1082 cm^−1^. The band at 1044 cm^−1^ was assigned to the C-CH_3_ stretching. The C-C stretching is denoted by the band at 868 cm^−1^. In the case of the PLA samples with capsules (CAF@UiO-66c), their corresponding bands also appear in the composite’s spectra, especially the bands corresponding to the MOF, the caffeine bands being not very noticeable. The bands corresponding to the UiO-66 present in the composite samples are at 1578 cm^−1^ (C=O) and 1507 cm^−1^ (phenyl ring C=C), and the band at 664 cm^−1^ is due to the (O-Zr-O) bond. Moreover, there are slight bands that could correspond to caffeine, specifically at 1700 and 1652 cm^−1^, which denote the presence of the carbonyl (C=O) bond. The results are in agreement with what was observed by XRD in terms of the preservation of the structure and functional groups of the MOF. For polymer composites with pure caffeine, bands corresponding to this additive are not visible in the spectra.

**Figure 10:**
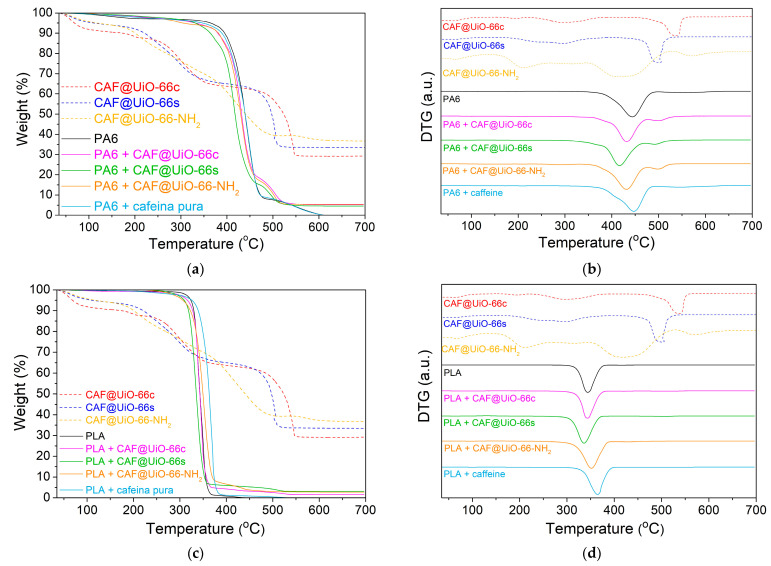
TGA (**a**) and DTG (**b**) characterization of PA6 composites. TGA (**c**) and DTG (**d**) characterization of PLA composites. TGA was carried out under air flow.

The prepared PA6 and PLA composites were characterized by TGA (Figure 10). The TGA curves of the pure PA6 composites (Figure 10a) show differences between the pure polymer and PA6 with capsules. In that of PA6, there is a small continuous degradation (<5 wt%) between 100 and 250 °C that may be related to unreacted reagents and small amounts of water. The large mass loss related to the degradation of the polyamide begins at around 350 °C with a maximum in DTG at 445 °C (Figure 10b). In the PA6 with capsules, the degradation of the sample occurs earlier than in the pure PA6, with a maximum in DTG at 432 °C, 420 °C, and 430 °C for UiO-66c, UiO-66s, and UiO-NH_2_, respectively. This faster degradation may be due to a slight catalytic effect of the MOF present in the capsules since the degradation of PA6 with pure caffeine has a maximum DTG (446 °C) at the same temperature as the pure PA6. In the polymer composites with capsules and pure caffeine, there is a detectable weight loss in the DTG before the degradation of the polymer, which could be related to the presence of caffeine as well as stabilization since it occurs at higher temperatures than pure and encapsulated caffeine. Furthermore, in the polymer composites with capsules, a clear weight loss is shown after the degradation of the polymer, which may be due to the MOF. This represents an acceleration in the degradation of the MOF, probably due to the caloric effects of the degradation of the polymer, which indicates good integration of the capsules in the polymer. It should be noted that, in these samples, the final residue of the TGA carried out in the air is ZrO_2_. 

Regarding the residual weight loss after each analysis, the amount of zirconium dioxide (zirconia) in the sample can be calculated. Therefore, the experimental percentage of capsules inside the composites can be determined. For the PA6 composites, the final percentage of ZrO_2_ was 5.21 wt%, 4.57 wt%, and 5.40 wt% for UiO-66c, UiO-66s, and UiO-66-NH_2_, respectively. Taking into account the previous values and UiO-66 as the molecular formula C_48_H_28_O_32_Zr_6_ and UiO-66-NH_2_ as C_48_H_28_N_6_O_32_Zr_6_, the experimental percentage of capsules in the composites has been calculated, as shown in Table 2. The theoretical percentage of capsules was 10 wt%. The difference between this value and the previous ones may be due to the small quantity of sample used in the TGA analysis, which may not be very reproducible from the total sample.

Figure 10c,d shows little variation in the degradation of the PLA composites with and without UiO-66 capsules. In the sample with pure caffeine, there is a certain delay in the degradation of the polymer. In the samples with capsules, there is a very slight advance in the degradation of the polymer. In any case, a residual weight was observed after each analysis, reaching values of 1.56 wt% (composite with UiO-66c), 3.11 wt% (composite with UiO-66s), and 2.75 wt% (composite with UiO-66-NH_2_). With these data, as with the PA6 composites, the percentage of capsules in the PLA composite was determined (Table 2). The values, although they are of the expected order, do not agree with the theoretical percentage, which is related to the small amount of sample used in the analysis.

**Figure A5:**
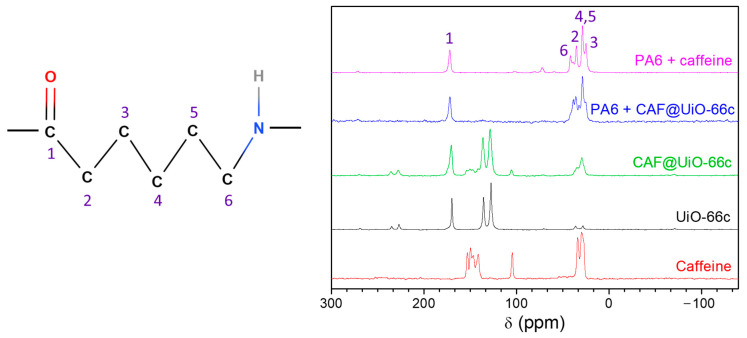
^13^C NMR spectra of PA6 composites compared with the solid sample’s spectra.

To confirm the presence of caffeine in the polymer composites, a ^13^C NMR analysis (Figure A5) was also performed of the PA6 composites with caffeine and with CAF@UiO-66c, the sample which gave the best release results, as will be shown below. The ^13^C NMR spectra of PA6 can present different forms depending on the polyamide structure [68]. The PA6 composite spectra obtained with caffeine and capsules are similar to the spectra of pure PA6. Neither caffeine nor UiO-66 were detected inside the composites.

**Figure 11:**
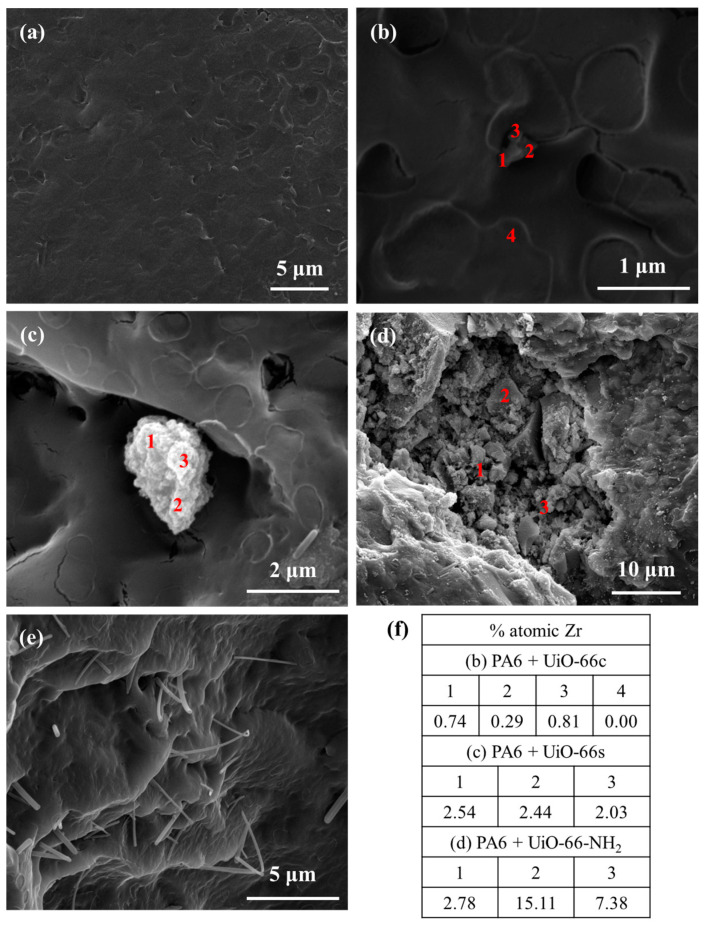
SEM images of PA6 composites (**a**) pure PA6, (**b**) PA6 + CAF@UiO-66c, (**c**) PA6 + CAF@UiO-66s, (**d**) PA6 + CAF@UiO-66-NH_2_, (**e**) PA6 + caffeine, (**f**) Atomic percentages of Zr in cross-section SEM images obtained from EDX analysis.

**Figure 12:**
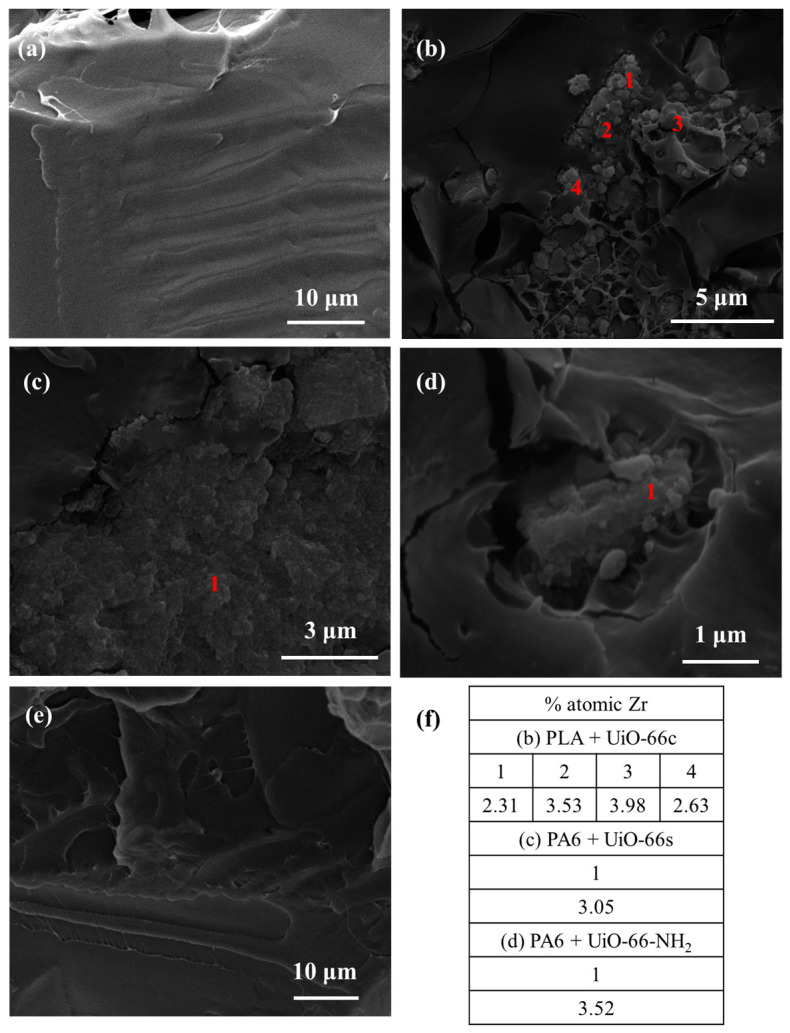
SEM images of PLA composites (**a**) pure PLA, (**b**) PLA + CAF@UiO-66c, (**c**) PLA + CAF@UiO-66s, (**d**) PLA + CAF@UiO-66-NH_2_, (**e**) PLA + caffeine, (**f**) atomic percentages of Zr in cross-section SEM images obtained from EDX analysis.

Figure 11 and Figure 12 show the SEM images of the PA6 and PLA composites. As expected, for the pure PA6 composite no particle was observed (Figure 11a). In the composites with CAF@MOF, UiO-66 particles were seen in the PA6 composite cross-section (Figure 11b–d) with a shape very similar to the particles before being added to the composite and showing good contact between the capsules and the PA6. This would be related to the organic character of the MOF that makes it compatible with the polymer. In general, regardless of the type of capsule, both isolated particles (Figure 11b) and particle aggregates (Figure 11c,d) can be found in the samples, although in the sample with UiO-66c, the particles are less aggregated. On the samples with capsules (Figure 11b–d), EDX analysis was performed at the indicated points. The Zr atomic percentage values of the EDX analysis of these composites are shown in Figure 11f. In the PA6 + CAF@UiO-66c sample, the Zr element was detected by EDX analysis at different points of the solid particle. Another zone away from this particle was also analyzed (point 4), where the Zr percentage was zero. In the composite sample with synthesized UiO-66, zirconium was also detected in an aggregate of particles reaching values around 2.5 at%. If a larger aggregate of particles is analyzed, such as the one in the composite of UiO-66-NH_2_ capsules, zirconium is detected in greater quantities (Figure 11f). In any case, with the EDX analysis and the preservation of its shape, it is possible to confirm the presence of the MOF after the composite manufacturing process. It should be noted that the composite manufacturing process occurs at 260 °C, a temperature which, as has been observed in the TGA, the MOF capsules with caffeine can withstand. Figure 11e clearly shows the caffeine crystals inside the PA6 composite, which indicates that the pure caffeine added to the composite is also capable of withstanding the processing conditions, as was already intuited in the TGAs of the composites.

In the PLA composites with capsules (Figure 12b–d), MOF particles were also observed preserving their appearance, showing good contact with the polymer, and sometimes forming aggregates. In addition, the presence of the Zr element was confirmed by EDX analysis, reaching values between 2–4 at%. It should be noted that the processing of PLA composites was at 190 °C, which is slightly lower than the processing of PA6 (260 °C) in which, as mentioned above, the capsules were not degraded. Unlike the PA6 + caffeine composite, for the PLA + caffeine sample, no caffeine crystals were observed in the cross-section. This may be due to the reaction that takes place between PLA and caffeine, which is explained in more detail in the caffeine delivery section.

The melt volume rate values of the PA6 and PLA composites were measured during composite preparation, and the values are represented in Table 3. Composites with the capsules, especially PLA, presented lower fluency rates in comparison with the pure polymers. This decrease is not very pronounced, so it does not affect the composites’ processing or their mechanical properties. The values for the PLA composites are particularly low with respect to the PA6 composites due to the lower fluidity of the pure polymer.

In summary, it can be stated that the incorporation of the capsules with caffeine in the PA6 and PLA composites has been successful given the following evidence obtained in the characterization of the composite materials: the XRD patterns show the preservation of the structure of the capsules; FTIR analysis denotes the presence of the main functional groups of the capsules; TGA reveals a certain influence in the degradation of the composite due to the presence of the capsules observing a final residue related to them; the SEM images show that the shape of the capsules is preserved with good contact with the polymers; and the chemical analysis by EDX analysis in the SEM images shows the clear presence of zirconium. Furthermore, as will be shown in the following section, caffeine is progressively released from the polymer composites.

### 3.4. Caffeine-Release

#### 3.4.1. CAF@MOF

**Figure 13:**
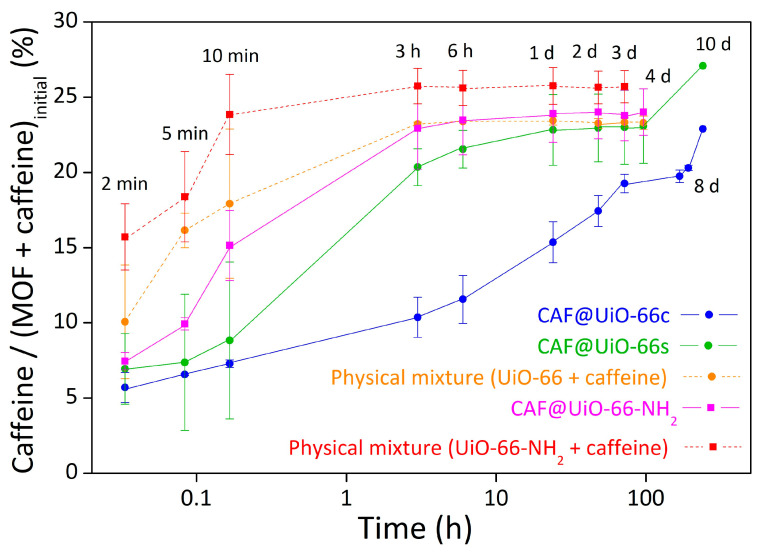
Release experiments of solid samples, CAF@UiO-66c, CAF@UiO-66s, CAF@UiO-66-NH_2_, and a physical mixture of both MOF with caffeine, determined by UV–Vis absorption. The error bars come from the measurement of two samples prepared in different batches.

Figure 13 shows the evolution with time of caffeine release in the CAF@MOF samples. As shown in the figure, the best encapsulation result was achieved for the CAF@UiO-66c sample since the release is more gradual than for the other samples. With this sample, the maximum caffeine percentage was reached at seven days until a value of 20 wt%. For the CAF@UiO-66s sample, the initial release is similar to the CAF@UiO-66c, but at shorter times the release is faster, achieving a value of around 20 wt% at 3 h. Then, for longer times, the value stabilizes at four days (23 wt% of caffeine in the capsules). This difference can be attributed to the textural properties of UiO-66. On the one hand, due to the smaller particle size of the UiO-66s than the UiO-66c, the external surface in contact with water is higher, so the release would be faster. On the other hand, the specific surface area of the UiO-66s is lower and with a smaller volume of micropores, so its capacity is more limited than the UiO-66c. Although UiO-66s has mesopores, these appear to release caffeine more quickly. Furthermore, as seen by TGA, there appear to be two types of caffeine in this sample and in general less interaction with the MOF than in the CAF@UiO-66c sample. In these release experiments, the temperature was increased to 80 °C for two days (from day 8 to day 10), in order to know the maximum caffeine content in the capsules, and reached values of 23 wt% and 27 wt% for UiO-66c and UiO-66s, respectively. In both cases, the percentages were similar to those calculated by TGA and, therefore, also to the theoretical value of encapsulated caffeine (25 wt%). For the physical mixture, the release was faster in all cases, since at 10 min the caffeine release was around 18 wt%. In both CAF@UiO-66, there was an improvement in the rate of caffeine release related to the physical mixture. This difference was more significant for UiO-66c, as already mentioned.

The results for UiO-66-NH_2_ showed an improvement in the caffeine release for the sample encapsulated in the ball mill (CAF@UiO-66-NH_2_) compared to the physical mixture (caffeine + UiO-66-NH_2_). Comparing the ball mill samples, the synthesized MOFs (UiO-66s and UiO-66-NH_2_) presented similar results. These release results were in accordance with the surface area and TGA results, which are very similar. At low times, the release of caffeine is higher for UiO-66-NH_2_, which may be due to the amino group hindering caffeine encapsulation, so the caffeine was not well encapsulated. These results are in agreement with observations in the literature that the −NH_2_ group causes competitive adsorption of the water used as a medium for impregnation, which worsens the encapsulation of caffeine [69].

#### 3.4.2. Polymer Composites

**Figure 14:**
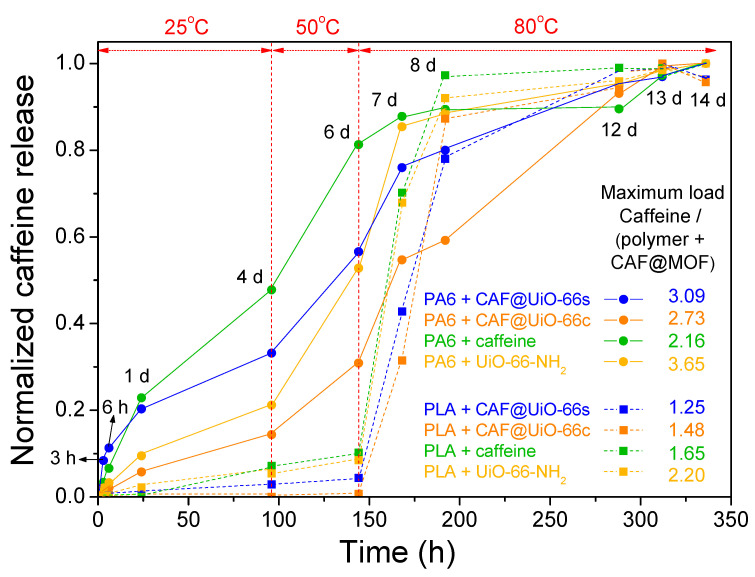
Normalized values of caffeine release in PA6 and PLA composites showing the evolution with time and temperature. The amount of polymer composites introduced was 0.5 g for all samples.

The caffeine delivery from the polymer composites was also studied. Samples were put in contact with distilled water for a period of time (until 14 days) and the temperature was increased from 25 °C to 80 °C. The polymer composites were maintained for several days at this final temperature, until reaching a constant value of caffeine in the solution. Figure 14 shows a comparison of the release results of both kinds of polymers with all the caffeinated composites. Since the mixing method used for composite processing did not produce a homogeneous distribution of the capsules and the polymer, as observed by SEM, the results obtained, given the small amounts of composites taken in the release, will depend on the pieces of composite selected to carry out the release. The values in Figure 14 have been normalized taking into account the maximum value obtained in each composite sample, which in most cases corresponds to the final value (14 days) already stabilized over time.

For PA6, the release is faster in the polymer composite containing caffeine without encapsulation. Therefore, the encapsulation process in the UiO-66 makes the release of caffeine more gradual over time, extending the service life of the textile product. Comparing the PA6 polymer composites with the capsules, the ones prepared with UiO-66c exhibit more gradual caffeine release, as occurred with the capsules prepared with this MOF (see Figure 13). As indicated, the sample with UiO-66c, which has the largest surface area, microporosity, and particle size, and in the TGA analysis delays the degradation of caffeine indicating a deeper encapsulation, shows the most gradual release. In the case of the sample with UiO-66-NH_2_, the release of caffeine seems slower than for UiO-66s in the PA6 composite but much faster than UiO-66c. In any case, it seems that the textural properties have a more prominent role in the release than the presence of the amino group.

In the case of the PLA composites, the release was very slow at initial times when the temperature was relatively low and experienced a sharp increase when the temperature was increased up to 80 °C. Studies in the literature have analyzed the effect of basic drugs, such as caffeine [44], on PLA degradation. They have concluded that basic drugs neutralize the carboxyl end groups and minimize the autocatalytic effect of acidic chain ends in polymer degradation and, therefore, the drug release rate decreases [70]. Furthermore, the release of caffeine is delayed when it is encapsulated, in this case being evident at high temperatures. In any case, it seems that an interaction is established between PLA and caffeine, which means that even the caffeine sample without encapsulation shows a remarkably slow release. This interaction between caffeine and PLA could occur during extrusion.

**Figure A6:**
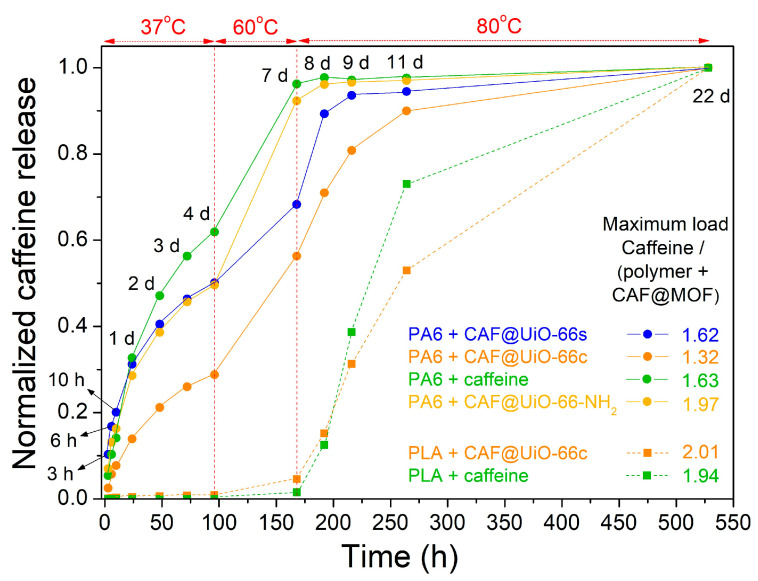
Normalized values of caffeine release in PA6 and PLA composites showing the evolution with time and temperature. The amount of composites introduced was 7.956 g, 8.414 g, 7.398 g, and 7.981 g for PA6 composites (UiO-66c, UiO-66s, caffeine, and UiO-66-NH_2_) and 9.380 g and 9.4261 g for PLA composites (UiO-66c and caffeine).

To verify the reproducibility of the trends obtained, an experiment similar to the previous one was repeated with some representative samples but with a greater amount of composite, between 7.4–9.4 g (Figure A6). In this experiment, it can be observed again that in the PA6 polymer, the encapsulation of caffeine delays its release, with the CAF@UiO-66c capsules showing the slowest release. In the PLA polymer, it is observed that there is a differential delay in the release of caffeine with respect to the PA6 polymer. In PLA at low temperatures, it does not seem that the encapsulation of caffeine favors its retention, and high temperatures are needed to clearly show that the encapsulation in UiO-66c delays its release. These results suggest that an interaction is established between caffeine and PLA that could occur during extrusion and that undoubtedly delays its release. In any case, for the PA6 composites, the encapsulation in UiO-66 is essential in order to achieve a more gradual release.

**Figure A7:**
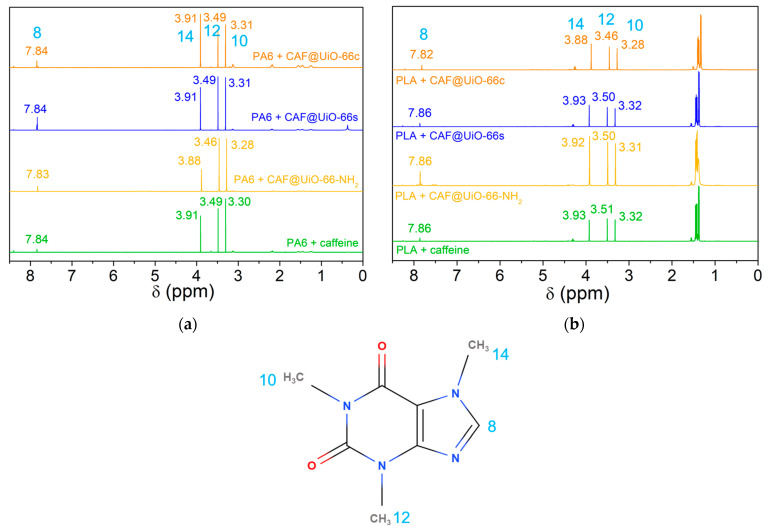
^1^H NMR spectra of liquids obtained after liquid–solid extraction at different temperatures (25–50–80 °C) at 12 days (80 °C): (**a**) PA6 composites. (**b**) PLA composites.

To confirm the presence of caffeine, the liquids obtained from the solid–liquid extractions shown in Figure 14 (at 12 days) were analyzed by ^1^H NMR (Figure A7). The ^1^H NMR spectrum of caffeine consists of the sole ring ^1^H signal (7.58 ppm) and the signals from three methyl groups (3.37, 3.55, and 4.01 ppm) [71]. In Figure A7a (PA6) and A7b (PLA), peaks can be observed corresponding to caffeine in the ^1^H NMR spectra of the polymer composites. For the PA6 composites, there are some little peaks apart from these, which may be due to polyamide decomposition. In the case of the PLA composites, peaks other than caffeine appear with higher intensity, due to the fact that polylactic acid at 80 °C decomposes more than polyamide. These peaks appear in other regions than the caffeine ones, therefore they are not influenced by the presence of PLA or PA6.

### 3.5. Benchmark

The use of UiO-66 in encapsulation has been widely reported by other authors due to its great adsorption properties and high thermal stability. The encapsulated compound was caffeine in several studies, among other additives. Sarker et al. [34] reported the storage and controlled release of caffeine from UiO-66 and the functionalized form UiO-66-COOH, achieving caffeine loadings of 21.4 and 17.5 wt%, respectively. The release of caffeine from pristine UiO-66 studied in a buffer solution (PBS, 0.01 M, pH: 7.4), was fast and delivered 85% of the encapsulated caffeine in 12 h. Cunha et al. [32] achieved a caffeine loading of 22.4 wt% and 13.2 wt% for UiO-66 and UiO-66-NH_2_, respectively. In a later work, they analyzed the caffeine delivery of these MOFs in aqueous media at 37 °C. The experiments achieved 75% and 60% caffeine release in 24 h for the UiO-66 and amino-functionalized form, respectively [33]. Zhou et al. reported the encapsulation of caffeic acid (CA) in UiO-66, improving its stability. CA@UiO-66 showed the highest CA loading rate of 56% and a release ability of CA of 83% until 100 h [72]. Caffeine was also encapsulated in UiO-66 by solvent-free encapsulation under high-pressure contact of the additive and the MOF. The encapsulation value achieved with UiO-66 was 15 wt% [37]. Moreover, by a simple impregnation method, caffeine was encapsulated in UiO-66 and UiO-66-NH_2_ by Devautour-Vinot et al., obtaining encapsulation values of 22.4 wt% and 13.2 wt%, respectively [69]. In our study, 20–25 wt% of caffeine was encapsulated in the capsule (0.333 mg of caffeine/g MOF), which is slightly above the values mentioned in the literature. Although very dependent on the operational conditions, with the best material used in this work (UiO-66c) approximately 75% of the encapsulated caffeine was released in 24 h at room temperature, which is in line with the data discussed above.

In the production of PA6 fibers through extrusion, Pérez et al. [15] achieved the incorporation of α-tocopheryl acetate into porous materials, specifically zeolite Y. The resulting PA6 fibers contained 0.075 wt% of the additive. In a similar process, Zornoza et al. [16] encapsulated 0.03%, 0.06%, and 0.17 wt% of caffeine in zeolite Y, MIL-53, and ZIF-8, respectively. In our work with the UiO-66 MOF incorporated in PA6, a higher load of up to 2.5 wt% of caffeine was achieved. However, it must be taken into account that in the cited work the addition was performed during the spinning process to obtain fibers with diameters of 15–20 μm, while in our work melt extrusion was carried out to obtain composites of about 1 mm in diameter.

## 4. Conclusions

In the present work, composite materials of polymers and metal–organic frameworks based on zirconium (UiO-66) with encapsulated caffeine were successfully developed by extrusion. In particular, the following conclusions can be listed:

- As indicated by the different characterization techniques (XRD, FTIR, SEM, TGA, NMR, and nitrogen adsorption), UiO-66 has been prepared through a solvent-free synthesis with textural properties (specific surface area and pore size distribution) and a particle size that are different from those of commercially purchased UiO-66.
- With the same characterization techniques indicated in the previous point, it has been demonstrated that UiO-66-NH_2_ has been prepared through a synthesis with ethanol, avoiding the use of toxic solvents, such as DMF.
- The encapsulation of 25 wt% of caffeine by milling assisted by a small amount of water in the capsules (MOF@CAF) has been carried out correctly with the available UiO-66 and also with UiO-66-NH_2_. This assertion is supported by the comparison of MOF and caffeine with these capsules using various techniques, such as XRD, FTIR, SEM, TGA, NMR, and nitrogen adsorption.
- The caffeine release study of the MOF@CAF samples shows a much slower release compared to the physical mixture of caffeine with the MOF. The release is especially slow in sample UiO-66c.
- The PA6 + MOF@CAF and PLA + MOF@CAF composites have been prepared by extrusion with a theoretical caffeine load of 2.5 wt% and, despite the high processing temperatures (190 °C and 260 °C for PLA and PA6, respectively), the capsules retain their fundamental characteristics, as indicated by their characterization (XRD, FTIR, SEM, EDX, and TGA).
- The caffeine release study of the PA6 + MOF@CAF composites shows a much slower release compared to incorporating pure caffeine into the polymer. Therefore, the encapsulation of caffeine has a determining effect on delaying its release in PA6 polymers.
- Among the MOFs, UiO-66c, with its greater surface area, microporosity, and larger particle size, shows the slowest caffeine release in the polymeric composites, which is also in accordance with the release in the capsules.
- The amino group in the UiO-66 does not appear to play a prominent role in the release of caffeine.
- Both in the CAF@MOF capsules and in pure caffeine, the PLA polymer establishes an interaction with caffeine that delays its release markedly compared to the PA6 polymer. In the PLA composites, the effect of encapsulation is observed at high temperatures.

Finally, it should be mentioned that procedures respecting sustainability have been developed, so that the syntheses of the zirconium-based MOFs have been carried out avoiding the use of toxic solvents. Likewise, the encapsulation of caffeine in these synthesized MOFs has been carried out by liquid-assisted milling, considerably reducing the amount of solvents, reducing the process steps, and avoiding residues. Following the same trend, the MOF@CAF capsules have been embedded during the extrusion process in recycled polyamide 6 (PA6) and in a biopolymer based on polylactic acid (PLA) that is biodegradable. These developed composite materials are the basis for the improvement of sustainable functionalized textile fibers with embedded durable capsules that can dose additives, such as caffeine, slowly.

## Figures and Tables

**Table 1:** Textural properties of UiO-66c, UiO-66s, and UiO-66-NH_2_ before and after caffeine encapsulation.

	BET Surface Area (m^2^/g)	Total Pore Volume ^1^ (cm^3^/g)	Micropore Volume ^2^ (cm^3^/g)
UiO-66c	1298 ± 24	0.626	0.544
UiO-66s	771 ± 12	0.600	0.271
UiO-66-NH_2_	650 ± 11	0.380	0.240
CAF@UiO-66c	216 ± 3	0.130	0.078
CAF@UiO-66s	396 ± 6	0.340	0.129
CAF@UiO-66-NH_2_	150 ± 2	0.120	0.044

^1^ At P/P_0_ = 0.97. ^2^ Using t-plot.

**Table 2:** Experimental percentage of capsules inside the PA6 and PLA composites. The theoretical value is 10 wt% in all cases.

Polymer	Capsule	wt% Capsules (Experimental) ^1^
PA6	CAF@UiO-66c	9.4 ± 5.5
	CAF@UiO-66s	9.0 ± 6.7
	CAF@UiO-66-NH_2_	11.4 ± 8.0
PLA	CAF@UiO-66c	5.7 ± 1.5
	CAF@UiO-66s	11 ± 6.0
	CAF@UiO-66-NH_2_	8.9 ± 0.4

^1^ The value has been calculated by TGA with at least two samples.

**Table 3:** Melt volume rate values (MVR) of PA6 and PLA composites.

Polymer	Capsule	MVR (cm^3^/10 min)
PA6	Pure	78.78 ± 4.50
	CAF@UiO-66c	73.42 ± 6.37
	CAF@UiO-66s	60.32 ± 14.17
	CAF@UiO-66-NH_2_	63.88 ± 6.91
	Caffeine	77.01 ± 4.65
PLA	Pure	5.75 ± 0.45
	CAF@UiO-66c	2.40 ± 0.88
	CAF@UiO-66s	2.96 ± 1.45
	CAF@UiO-66-NH_2_	1.25 ± 0.36
	Caffeine	3.66 ± 0.19

## Data Availability

Complementary data are available as an appendix and upon request to the authors.

## Appendix A

**Table A1:** Particle size values (nm) obtained from Scherrer’s equation calculated at different diffraction peaks (2·θ) for UiO-66c, UiO-66s, and UiO-66-NH_2_.

	Particle Size (nm)		
2·θ	7.4°	8.5°	25.7°
UiO-66c	49.5	56.9	53.6
UiO-66s	13.4	21.9	23.5
UiO-66-NH_2_	40.2	42.4	43.6
